# Photoperiod Influences Growth and *mll* (Mixed-Lineage Leukaemia) Expression in Atlantic Cod

**DOI:** 10.1371/journal.pone.0036908

**Published:** 2012-05-09

**Authors:** Kazue Nagasawa, Alessia Giannetto, Jorge M. O. Fernandes

**Affiliations:** Faculty of Biosciences and Aquaculture, University of Nordland, Bodø, Norway; Kyushu Institute of Technology, Japan

## Abstract

Photoperiod is associated to phenotypic plasticity of somatic growth in several teleost species. However, the molecular mechanisms underlying this phenomenon are currently unknown but it is likely that epigenetic regulation by methyltransferases is involved. The MLL (mixed-lineage leukaemia) family comprises histone methyltransferases that play a critical role in regulating gene expression during early development in mammals. So far, these genes have received scant attention in teleost fish. In the present study, the mean weight of Atlantic cod juveniles reared under continuous illumination was found to be 13% greater than those kept under natural photoperiod conditions for 120 days. We newly determined cDNA sequences of five *mll* (*mll1*, *mll2*, *mll3a*, *mll4b* and *mll5*) and two *setd1* (*setd1a* and *setd1ba*) paralogues from Atlantic cod. Phylogenetic analysis revealed that the cod genes clustered within the appropriate *mll* clade and comparative mapping of *mll* paralogues showed that these genes lie within a region of conserved synteny among teleosts. All *mll* and *setd1* genes were highly expressed in gonads and fast muscle of adult cod, albeit at different levels, and they were differentially regulated with photoperiod in muscle of juvenile fish. Following only one day of exposure to constant light, *mll1*, *mll4b* and *setd1a* were up to 57% lower in these fish compared to the natural photoperiod group. In addition, mRNA expression of myogenic regulatory factors (*myog* and *myf-5*) and *pax7* in fast muscle was also affected by different photoperiod conditions. Notably, *myog* was significantly elevated in the continuous illumination group throughout the time course of the experiment. The absence of a day/night cycle is associated with a generalised decrease in *mll* expression concomitant with an increase in *myog* transcript levels in fast muscle of Atlantic cod, which may be involved in the observed epigenetic regulation of growth by photoperiod in this species.

## Introduction

Histone modifications, including acetylation, methylation, phosphorylation, and ubiquitination, have emerged as key mechanisms of transcriptional regulation and may serve as an epigenetic regulation marking system that is responsible for maintaining heritable programs of gene expression during ontogeny [1], [2]. In particular, histone methylation plays a critical role in gene expression and epigenetic regulation [3], [4]. Mixed-lineage leukaemias (MLLs) are histone methyltransferases (HMTs) that specifically methylate histone H3 at lysine 4 (H3K4) and are linked to gene activation [5], [6], [7]. In yeast, Set1 exists as a multi-protein complex (known as COMPASS), which is the only H3K4-specific HMT [8], [9]. In contrast, the human genome encodes seven Set1 homologues: MLL1 [10], MLL2 [11], MLL3 [12], MLL4 [13], MLL5 [14], SETD1A and SETD1B [15]. Each of these protein acts as a multi-protein complex sharing several common subunits [2].

MLLs are widely expressed during development and in most adult tissues, including myeloid and lymphoid cells [16]. Moreover, they are well known as master regulators of homeobox-containing (Hox) genes that are critical for cell differentiation and development [6], [17], [18]. Heterozygous *Mll1*-knockout mice show posterior shifts in *Hox* gene expression, and homozygous *Mll1*-knockout mice are embryonic lethals in which the patterns of *Hox* expression initiate normally but are not maintained past embryonic day 9.5 [19]. The involvement of MLL2 in mammalian myogenesis has been demonstrated by McKinnell et al. [20], who reported that an HMT complex containing MLL2 interacted with paired box protein 7 (Pax7) to directly regulate the expression of myogenic factors, particulary *myogenic factor 5* (*Myf-5*). MLL5 also regulates the cell cycle in cultured myoblasts and is required for the expression of trascription factors that regulate the myogenic programme, including *Myf-5* and *myogenin* (*MyoG*) [21]. The full repertoire of *mll* paralogues has never been determined in fish species so far and the few reports available are restricted to model fish species such as zebrafish, (*Danio rerio*) [5,22], except for a single *mll* gene that was cloned in tiger pufferfish (*Takifugu rubripes*) [23].

Atlantic cod (*Gadus morhua*) is one of the most economically important fish species worldwide. Nevertheless, the profitability of the cod farming industry is severely restricted by precocious sexual maturation of the fish in captivity, which reach puberty prior to attaining commercial size [24]. Photoperiod manipulation, typified by continuous illumination, has been successfully used to delay sexual maturation to some extent in Atlantic cod [24], similarly to what has been observed in other farmed fishes, including Atlantic salmon (*Salmo salar*) [25] and European sea bass (*Dicentrachus labrax*) [26]. While most studies involving photoperiod manipulation in Atlantic cod have been conducted on two-year old fish [24], it has been reported that short-term photoperiod manipulation during early juvenile stages has a significant positive effect on somatic growth, which is dependent on genetic background and environmental temperature [27]. Remarkably, juvenile cod kept under continuous light for three months had a significantly higher weight than the simulated natural photoperiod group and this difference remained even after 30 months of sea-pen rearing under identical ambient conditions until harvest. By this point, fish subjected to the initial continuous light treatment were up to 9% larger than their counterparts reared under natural photoperiod [28]. The present study was designed to further our limited understanding about the epigenetic regulation of somatic growth in Atlantic cod, with particular focus on the *mll* family, since these genes are known to play a crucial role in myogenesis. We have cloned all representatives of the *mll* gene family in Atlantic cod and examined their expression levels in fast muscle of juvenile fish kept under continuous illumination or simulated natural photoperiod.

**Figure 1:**
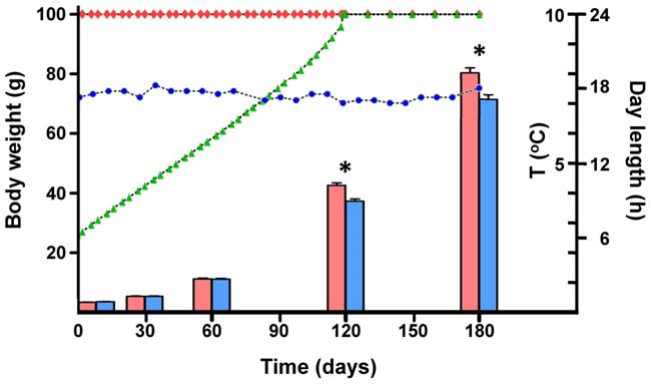
Growth history of Atlantic cod juveniles reared under continuous light (LD 24:0, red bars) or natural photoperiod conditions (LDN, blue bars) for six months. Details of the light regimes are shown by red diamonds or green triangles for LD 24:0 and LDN groups, respectively. Sea water temperature is also indicated by blue circles. Significant differences in mean weight between the two light groups at a particular time point (two-tailed t-test, n = 123) are highlighted by an asterisk.

## Results

### Influence of photoperiod on growth performance

The initial mean weight did not differ between fish in the continuous light and natural photoperiod groups (Fig 1, P>0.05, n = 123). Significant differences in mean weight between them were only observed at days 120 and 180 (P<0.0001 and P<0.0002, respectively, n = 123). Fish kept under continuous illumination were *circa* 13.3% and 10.5% larger than their counterparts in the natural photoperiod group at days 120 and 180, respectively (Fig 1).

**Table 1:** Gene name, GenBank accession number, primer sequences (5′ to 3′), amplicon sizes (bp) and PCR efficiency (%) of *mll,* s*et1d* and myogenic genes cloned in Atlantic cod.

Name	GenBank	Degenerate PCR		qPCR		Size	E
*mll1*	GU441836	Fw:	AGCCCAGCTCWRYAAGATWGAGAAG	Fw:	GACCAGCCTAAGATCCAGAGCCA	176	75.8
		Rv:	GAWGGCATCCAYTGYARATTCTGACA	Rv:	GACAAGATCTTCTCCCGCTCCTC		
*mll2*	GU441837	Fw:	TAARCACACCATGGTCATYGAGTAT	Fw:	ATCTACGAGGAGCAGAACCG	150	82.4
		Rv:	TAAGGGATCTTGTGCTGATCGTCCT	Rv:	CTCTCGGTCAAAGGTCACAA		
*mll3a*	GU441838	Fw:	TACGCGGCCAGAGACATAGAGAAGT	Fw:	CGAGTACATCGGAACCATCA	147	81.4
		Rv:	TGAACACAGCACCAAGACGAAGAGA	Rv:	ACGTACCTCGCAGGTCCTC		
*mll4b*	GU441839	Fw:	TSAARAGRGTRTCCWSYYTSTCTGRCCG	Fw:	CGAAGGTTGACTTCCTGGAG	176	78.8
		Rv:	TCACTKSYGATCTCAAAGCGYAGRTGTGG	Rv:	AGACCGTGAGCTGTCCGAGT		
*mll5*	GU441840	Fw:	TCGGCCTTGTGGATGCACTTRGATGT	Fw:	AGCAGACACCGCGTACCT	84	91.2
		Rv:	TGCTGCTGTAAATCTGGTGWGGGTA	Rv:	CTGGACTTCTCCACAACCAC		
*setd1a*	GU441841	Fw:	TCAGARYATCAGACAGATGGTGGCTGA	Fw:	CGGCAGCAGCTACCTATTC	117	91.2
		Rv:	TACGATCTTCTTCTGGGACTCGATGGT	Rv:	ATCACCTTGGCGTAGCAGTT		
*setd1ba*	HQ315825	Fw:	TAYGTDGGVCAGAAYATCMGACAGGT	Fw:	GGAGAAGCGCTACGAGGAAG	100	86.1
		Rv:	TCAGTTRGGATTRCAGCTGTGGTTGATGAA	Rv:	GCGGGCGAAGTTGCCGCACT		
*myog*	JQ582407	Fw:	CAGTGCCTDCCCTGGGCCTGCAAG	Fw	CGCTGAAGAGGAGCACCCTGATG	121	79.3
		Rv:	TCCCGTCTCAKTSTCCTGCTGGTTGAG	Rv	TCCTGCTGGTTGAGCGAGGAGAC		
*myf5*	JQ619514	Fw:	GTGGGCCTGCAAGGCCTGCAAGCG	Fw	GACCTGCTGCACGAGCAGGTGGA	140	
		Rv:	CCRGGGCTCTCSGGSGTGCAGGGCTG	Rv	TAGAGGGCGGTCACTTGCGGCCA		97.8
*pax7*	JQ619515	Fw:	GTTTCYCAYGGTTGCGTCTCCAA	Fw	CGTGTTGAGGGCCCGGTTTGGCA	131	98.0
		Rv:	TCRAASGCCTTCTCCAGCTCCTCC	Rv	CCTCGTCTGTGCGGTTGCCTTTA		
*actb*	EX739174			Fw:	TGACCCTGAAGTACCCCATC	162	84.7
				Rv:	TCTTCTCCCTGTTGGCTTTG		
*arp*	EX741373			Fw:	TGATCCTCCACGACGATGAG	113	89.1
				Rv:	CAGGGCCTTGGCGAAGA		
*eef1a*	EX721840			Fw:	CACTGAGGTGAAGTCCGTTG	142	79.1
				Rv:	GGGGTCGTTCTTGCTGTCT		
*ubi*	EX735613			Fw:	GGCCGCAAAGATGCAGAT	69	84.8
				Rv:	CTGGGCTCGACCTCAAGAGT		

Reference genes used are also indicated.

### The *mll* gene family in Atlantic cod

**Figure 2:**
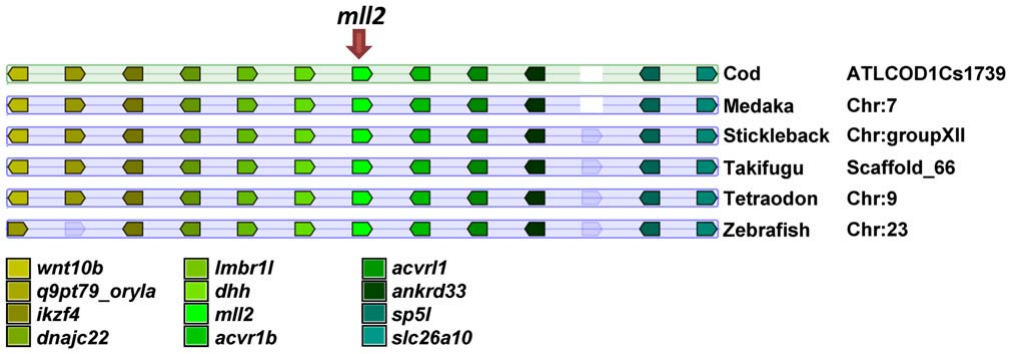
Partial synteny map of the genomic region surrounding *mll2*. Synteny was disrupted between teleosts and tetrapods. Orthologous genes in *Gadus morhua*, *Oryzias latipes*, *Gasterosteus aculeatus*, *Takifugu rubripes*, *Tetraodon nigroviridis* and *Danio rerio* are colour coded and represented by block arrows that show their orientation in the genome. *Mll2* paralogues are indicated by the arrow. Additional synteny results for other *mll* paralogues can be found in Figures S1–9.

Using degenerate PCR primers, we have successfully obtained partial cDNA sequences for five *mll* and two *setd1* paralogues in Atlantic cod: *mll1* (GU441836), *mll2* (GU441837), *mll3a* (GU441838), *mll4b* (GU441839), *mll5* (GU441840), *setd1a* (GU441841) and *setd1ba* (HQ315825) (Table 1). Comparative mapping of genes surrounding each *mll* and *setd1* paralogue showed that proximally-located genes lie within a region of conserved synteny amongst teleosts, namely Atlantic cod, medaka (*Oryzias latipes*), tiger pufferfish, zebrafish, stickleback (*Gasterosteus aculeatus*) and green-spotted pufferfish (*Tetraodon nigroviridis*) (Fig 2, Figures S1, S2, S3, S4, S5, S6, S7, S8, and S9). In contrast, synteny was disrupted between teleosts and tetrapods. Amongst all fish species examined, there was a particularly high degree of synteny conservation for *mll2* (Figure 2), *mll3b* (Figure S3) and *mll5* (Figure S6) genomic regions when compared to other *mll* paralogues. For example, ten genes (*wnt10b*, *q9pt79_oryla*, *ikzf4*, *dnajc22*, *lmbr1l*, *dhh*, *acvr1b*, *acvrl1*, *ankrd33 sp5l* and *slc26a10*) adjacent to *mll2* were positioned in the same order and orientation in equivalent regions amongst all teleost species examined (Fig 2). In contrast synteny was less conserved for setd1 paralogues (Figures S7, S8 and S9). Moreover, these analyses identified two paralogues of *mll3*, *mll4* and *set1d1b* in teleosts (Table S2, Figures S2 and S3, S4 and S5, S8 and S9, respectively).

**Figure 3:**
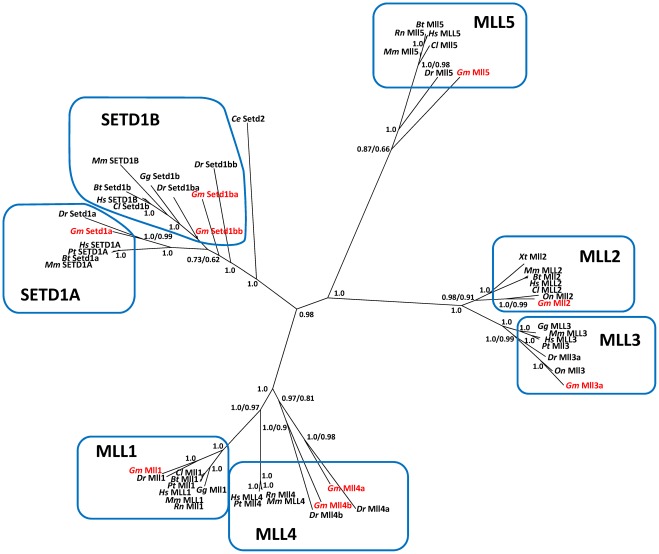
Phylogenetic tree of the seven *mll* and *setd1* paralogues found in vertebrates. Numbers at the nodes indicate posterior probability and approximate likelihood-ratio values obtained from Bayesian (left) and maximum likelihood (right) methods, respectively. Species abbreviations are as follows: *Bt, Bos Taurus; Ce, Caenorhabditis elegans; Cl, Canis lupus familiaris; Dr, Danio rerio; Gg, Gallus gallus; Gm, Gadus morhua; Hs, Homo sapiens; Mm, Mus musculus; On, Oreochromis niloticus; Pt, Pan troglodytes; Rn, Rattus norvegicus; Xt, Xenopus tropicalis.* GenBank accession numbers for *mll* sequences are listed in Table S1.

Deduced amino acid sequences of *mll* and *setd1* paralogues were obtained from the Atlantic cod genome sequence: Mll1 (2,470 amino acids, 62% covering full-length human MLL), Mll2 (1,991 aa, 36% covering full-length human MLL2), Mll3a (2,785 aa, 57% covering full-length human MLL3), Mll4a (744 aa, 27% covering full-length human MLL4), Mll4b (2,925 aa, 78% covering full-length zebrafish Mll4b), Mll5 (847 aa, 46% covering full-length human MLL5), Setd1a (744 aa, 33% covering full-length zebrafish Setd1a), Setd1ba (511 aa, 28% covering full-length zebrafish Setd1ba) and Setd1bb (134 aa, 18% covering full-length zebrafish Setd1bb). *Circa* 21% of the original 7936 positions were included in the alignment used for phylogenetic analysis. Bayesian phylogenetic reconstruction of the *mll* gene family is shown in Figure 3. *Mll* and *setd1* genes were clearly separated in seven clades that comprised *mll1*, *mll2*, *mll3*, *mll4*, *mll5*, *setd1a* and *setd1b* genes. The topology of the tree indicated a close association between *mll1* and *mll4* genes, as well as between the *mll2* and *mll3* clades. S*etd1a* and *setd1b* clusters were also more closely related to each other than to any other group. Atlantic cod *mll* genes cloned in this study clustered with the appropriate vertebrate *mll* homologues and were most closely related to their counterpart orthologues in zebrafish, as expected. Importantly, this phylogenetic tree clarified the paralogy of cod *mll* genes, showing that cod *mll3*, *mll4* and *setd1b* corresponded in fact to *mll3a*, *mll4b* and *setd1ba*, respectively.

### Tissue distribution of *mll* paralogues

**Figure 4:**
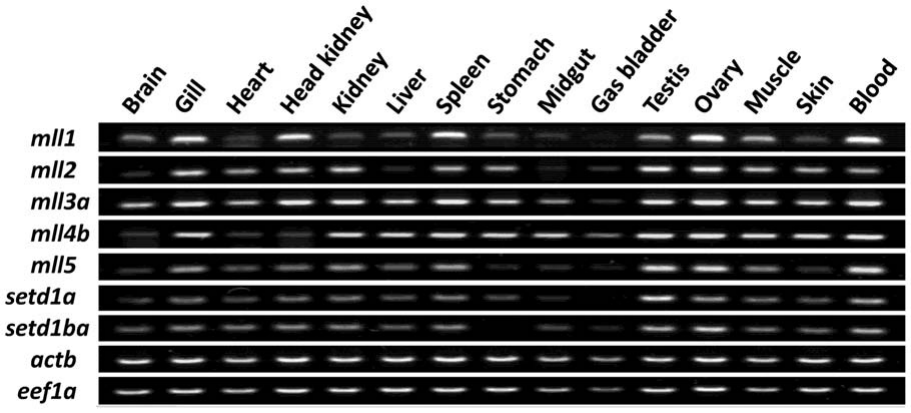
Representative tissue distribution of *mll* paralogues in adult Atlantic cod. cDNAs from various tissues (brain, gill, heart, head kidney, kidney, liver, spleen, stomach, mid gut, gas bladder, testis, ovary, fast skeletal muscle, skin and blood) were used for semi-quantitative RT-PCR. *Actb* and *eef1a* were used as endogenous references. Expression patterns were determined using three biological replicates.

With few exceptions, *mll* paralogues were ubiquitously expressed in all tissues examined, albeit at different levels (Fig 4). *Mll3a* transcripts were abundant in all tissues, except gas bladder, whereas *mll4b* was present in smaller amounts in brain, heart and head kidney. *Mll1*, *mll2*, *mll5*, *setd1a* and *setd1ba* paralogues were expressed at lower levels in the digestive tract (stomach and mid gut) and gas bladder. It is noteworthy that all seven *mll* and *setd1* paralogues were highly expressed in testes, ovaries, blood and fast skeletal muscle of adult cod.

### Differential expression of *mll* and key myogenic genes with photoperiod manipulation

**Figure 5:**
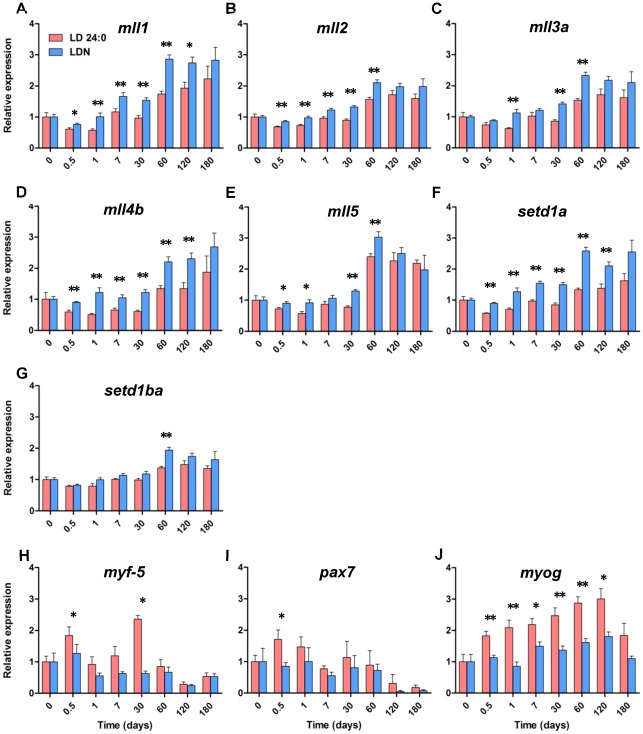
Quantification of *mll* paralogues and key myogenic genes (*myog*, *myf5* and *pax7*) in fast muscle of Atlantic cod juveniles reared under continuous light (red bars, LD 24:0) or natural photoperiod conditions (blue bars, LDN) for 6 months. In general, *mll* genes were differentially expressed between the two light groups and there was a decrease in *mll* transcript levels with continuous illumination as early as 12 hours, compared to the natural photoperiod group. Myog transcript levels were consistently higher in the constant light group compared to natural photoperiod. Asterisks * and ** indicate significant differences at p<0.05 and p<0.01, respectively (n = 6).

Relative *mll* expression in fast muscle of juvenile cod subjected to different photoperiod conditions was determined by qPCR, using the geometric average of *arp* and *ubi* reference genes to normalise the data. In general, all *mll* and *setd1* genes were significantly down-regulated at most time points in fast muscle of fish from the continuous light group (P<0.05, Fig 5). *Mll1*, *mll2*, *mll4b* and *setd1a* expression were significantly repressed in the continuous illumination group throughout the time course of the experiment until 120 days (P<0.05). Constant light was also significantly associated with a decrease in *mll3a*, *mll5* and *setd1ba* expression only at some time points from one to 60 days (P<0.05). Remarkably, there was a rapid change in *mll1*, *mll2*, *mll4b, mll5* and *setd1ba* expression with light regime, since their transcript levels were significantly lower in the continuous illumination group just 12 hours through the experiment. At day one, transcript levels of *mll1*, *mll3a*, *mll4b* and *setd1a* in fast muscle of fish from the continuous light group were reduced between 42 and 57% compared to the natural photoperiod group (Fig 5). *Mll* expression differences faded after 180 days, as expected since the light regime was identical for both groups (Figs 1 and 5). In addition, relative expression of myogenic regulatory factors (MRFs: *myog* and *myf5*) and *pax7* in fast muscle was examined in relation to photoperiod (Fig 5). Constant illumination was generally significantly associated with an increase in *myf5* and *pax7* expression, and the difference amongst light groups was significant at 12 hours (P<0.05). *Myog* transcript levels were significantly elevated with continuous illumination compared to the natural photoperiod group throughout the time course of the experiment from 12 hours until 120 days (P<0.05).

## Discussion

In the present study we have cloned *mll1*, *mll2*, *mll3a*, *mll4b*, *mll5*, *setd1a* and *setd1ba* orthologues in Atlantic cod and correlated their expression with differences in growth between fish reared under two photoperiod regimes. At days 120 and 180, age 1 Atlantic cod juveniles kept under continuous illumination were 13.3% and 10.5% larger than the ones from the natural photoperiod group, respectively. A similar effect has been previously shown in Atlantic cod juveniles [28], [29] but a recent report described a negative influence of photoperiod on growth rate [30]. This apparent discrepancy is most likely due to differences in the fish genetic background. There is a significant interaction between genotype and the response to photoperiod treatment. For example, specific growth rates of cod juveniles with the haemoglobin genotype *Hb-I(2/2)* increase from 1.8% day^−1^ under natural photoperiod to 2.3% day^−1^ under constant illumination at 13°C, whereas the average specific growth rate of *Hb-I(1/1)* fish remains almost unchanged with light regime [27]. It is not entirely clear how light stimulates somatic growth but it is not due to a simple extension of foraging activity and corresponding feed intake. In fact, in their natural environment age 1 Atlantic cod, like the ones used in our study, preyed preferentially on benthos at night time [31]. Moreover, our experimental fish were fed equal amounts daily and there were no apparent differences in feed consumption between the two light groups. It is likely that the short-term photoperiod treatment induces metabolic changes that promote higher growth rates, probably due to more efficient nutrient utilisation. Day length in Bodø, Norway, had reached 24 hours by 120 days into the experiment and, therefore, all fish were kept under identical conditions from this point onwards. Nevertheless, there was still an average 10.5% weight difference between the natural photoperiod and continuous illumination groups at 180 days. This indicates that short-term light manipulation may have a persistent effect on muscle growth and corroborates a previous report, which showed that juvenile cod reared under continuous light for three months and then transferred to sea pens became up to 9% larger than their counterparts initially kept under simulated natural photoperiod conditions [28].

Photoperiod has long been known as a factor affecting somatic growth in teleosts and it has been used in aquaculture to control growth and maturation of several commercial fish species [24], [25], [26]. However, the molecular mechanisms underlying growth plasticity induced by light are still unknown. Four basic helix-loop-helix (bHLH) transcription factors (*myoD myog myf5* and *myf6*) known as MRFs have received considerable attention as key players involved in determination and differentiation of skeletal muscle. Recent evidence supports the existence of interactions between MRFs and chromatin modifying complexes, including HMTs [20], [32]. Therefore we hypothesised that HMTs may be involved in this epigenetic regulation of growth in teleosts, since histone methylation is acknowledged as one of the most important systems to regulate chromatin status in mammals. In particular, MLL proteins are major H3K4-specific HMTs that regulate expression of Hox [18] and MRFs [21] during early development. MLL1 is known to catalyse H3K4 methylation of *HoxA7*, *HoxA9* and *HoxC8* [18], [33] and heterozygous *Mll1*-knockout mice (Mll1^+/−^) show impaired development due to insufficient Hox protein concentrations [19]. Microarray hybridisation studies have revealed that MLL1 affects the expression of 197 potential target genes in mice, namely cathepsin C and the CD34 stem cell antigen [34].

The role of HMTs, including MLLs, is still largely unclear in teleosts. To investigate their potential involvement in epigenetic regulation of muscle growth, we have cloned seven *mll* and *setd1* orthologues in Atlantic cod. Phylogenetic and synteny analyses revealed that unlike tetrapods most fish species contained two copies of *mll3*, *mll4* and *setd1b*. Their chromosomal localisation shows that these paralogues arose from the teleost-specific genome duplication prior to divergence of the teleost/tetrapod lineage [35]. Interestingly, *mll3a* is further duplicated in green-spotted pufferfish.

Cod *mll* paralogues had a broad tissue distribution in adult fish, albeit at various levels. Some *mll* genes (e.g., *mll1* and *mll5*) were highly expressed in blood, which might have biased the results observed in extensively vascularised tissues. *Mll2* and *mll3a* tissue distributions were similar to their human counterparts [11], [12]. In mice, *mll2* is required for development and spermatogenesis, and conditional knock-out male mice lacking *mll2* are infertile [36]. The high transcript levels of *mll2* and all other *mll* and *setd1* orthologues in Atlantic cod gonads indicate that they may play an important role in gametogenesis.

Differential expression in fast muscle with photoperiod was observed to some extent in all cod *mll* and *setd1* paralogues. The influence of light on *mll* transcript levels was noticeable as early as 12 hours following photoperiod manipulation, suggesting that *mll* genes may be associated with physiological adaptation to light and perhaps even involved in circadian rhythmicity. The largest differences in *mll* mRNA levels between photoperiod groups were detected at day one. By this point *mll1*, *mll3a*, *mll4b* and *setd1a* expression in fast muscle of cod from the continuous light group were reduced by up to 57% compared to the natural photoperiod group. *Mll2* was found to be down-regulated with continuous illumination at various time points from 12 hours to 60 days. There are no published functional or expression studies of *mll2* in teleosts but it is known to influence expression of key myogenic genes in mammals. In mice, overexpression of *Pax7* in satellite cells is known to result in elevated levels of *Myf5* expression [20]. The Wdr5-Ash2L-MLL2 HMT complex interacts directly with Pax7. This MLL2-Pax7 complex then binds to Myf5, resulting in H3K4 tri-methylation of surrounding chromatin [20]. Cod *pax7* was found to be significantly up-regulated in fast muscle with continuous illumination at 12 hours, even though *mll2* expression was slightly reduced. *Myf-5* expression in fast muscle, which might be induced by Pax7, was also elevated in the continuous light group at 12 hours and 30 days. Also, lysyl oxidase-like 1 (*loxl1*) is down-regulated 23-fold in *mll2*-knockdown HeLa cells [37]. LOX proteins are involved in collagen cross-linking and, therefore, play an important role in the structural integrity of muscle fibres [38]. In *mll5*-knockdown mice myoblast cell lines, expression of key players in myogenesis such as Pax7, Myf5 and Myog is impaired and these cells have limited ability to differentiate [21]. It seems that *mll5* controls the inappropriate expression of proliferation genes and maintains expression competence of key genes associated with myogenic differentiation in quiescent myoblasts. Throughout the time course of the our trial, *mll5* transcript levels were 20, 38, 40 and 31% lower in the continuous light group at 12 hours, one, 30 and 60 days, respectively. Down-regulation of *mll2* and or *mll5* in cod exposed to continuous illumination may result in a higher number of proliferating myoblasts, which would increase growth potential and explain at least in part the higher growth rate observed in these fish group compared to the natural photoperiod group. These results are consistent with the observed increase in transcript levels of *pax7* and *myf5* in fish kept under continuous illumination, since Pax7 is a known marker of myosatellite cells that is crucial for cell proliferation and Myf5 is involved in commitment of myoblasts to the myogenic programme [20]. Moreover, *myog* expression was consistently higher in the continuous light group compared to natural photoperiod throughout from 12 hours until 120 days. Myog plays a major role in myoblast differentiation and is known to be involved in thermally-induced phenotypic plasticity of muscle growth in fish [39].

We have characterized all representatives of the *mll* gene family in Atlantic cod and found that continuous illumination led to growth enhancement, which was accompanied by an increase in *pax7*, *myf5* and *myog* expression but associated with transcriptional repression of *mll* and *setd1* genes in fast muscle. To the best of our knowledge, this is the first study that investigated the molecular mechanisms of photic-induced plasticity of muscle growth in teleosts. MLL proteins are deemed global activators of multiple transcription factors and their reduced expression with light may be involved in epigenetic regulation of growth. For example, a decrease in activation of genes that inhibit myoblast differentiation into mature muscle fibres, such as *myostatin*, may induce enhanced growth of cod juveniles reared under continuous illumination. In zebrafish, knock-down of *myostatin-1* during embryonic somitogenesis results in up-regulation of muscle-specific transcription factors, including *myog* [40]. During the last two months of our photoperiod manipulation experiment light conditions were identical for both fish groups but the growth effect persisted, even if not accompanied by differential *mll* expression. Hence, epigenetic transcriptional memory may be due to chromatin remodelling that occurred during the first four months in response to photoperiod changes.

## Materials and Methods

### Photoperiod experiment and sample collection

Atlantic cod juveniles with an initial mass of 2.7±0.8 g (mean ± standard deviation [SD], n = 123) were kept at Mørkvedbukta Research Station (University of Nordland, Norway) in two groups of three 250 m^3^ tanks at an initial density of 130 individuals per tank and acclimated under continuous light until the start of the treatment. Sea water was pumped from 200 m depth and supplied at 7.4±0.4°C (mean ± SD). A commercial diet (Amber Neptun, Skretting AS, Stavanger, Norway) corresponding to 5% (w/w) of the fish body weight was provided daily by automatic belt feeders. Fluorescent white light tubes (Aura Light International AB, Karlskrona, Sweden) were used to illuminate the tanks evenly. Light intensity was monitored regularly with a Hanna Hai 97500 Luxmeter (Hanna Instruments, Kungsbacka, Sweden) and it was approximately 120 Lux near the water surface in the centre of the tanks. During the photoperiod experiment one group of three tanks was kept under continuous light whereas the other was kept under normal light regime that corresponded to natural environmental photoperiod conditions in Bodø (67°N), Norway from January until July 2010. *Circa* 120 fish from each group were weighed at the start of the experiment and then 0.5, 1, 7, 30, 60, 120 and 180 days thereafter. Statistical differences in mean weights were determined by Student's t-test using GraphPad Prism (GraphPad software, San Diego, USA). At each time point, 9 fish were humanely killed by immersion in seawater containing 1 g·L^−1^ tricaine methanesulfonate (Sigma, Oslo, Norway). Fast muscle was carefully dissected below the second dorsal fin from these specimens, taking special care to avoid skin and red muscle, and samples were snap-frozen in liquid nitrogen and stored at −80°C until RNA extraction.

Two-year old Atlantic cod were maintained in land-based tanks at Mørkvedbukta Research Station. Six fish with 50.8±3.8 cm fork length and 1.52±0.33 kg body weight were humanely killed as above. Brain, blood, gill, gas bladder, heart, liver, head kidney, kidney, stomach, mid gut, spleen, testis, ovary, muscle and skin were collected, snap-frozen in liquid nitrogen and stored at −80°C for subsequent RNA extraction. All procedures were conducted in accordance to the guidelines set by the National Animal Research Authority (Forsøksdyrutvalget, Norway) and approved by the Faculty of Biosciences (University of Nordlad, Norway) ethics committee.

### Cloning *mll* genes in Atlantic cod

Total RNA was extracted from the above adult cod tissues and used to synthesise cDNA with the QuantiTect kit (Qiagen, Nydalen, Sweden), as reported [41]. To identify *mll* paralogues in Atlantic cod, PCR amplification was performed with degenerate primer sets that were designed against the most conserved regions of each *mll* fish orthologues (Table 1). PCR reactions were performed using the Expand High Fidelity PCR System (Roche, Mannheim, Germany) with the following thermocycling conditions: initial denaturation at 94°C for 3 min, 35 cycles of amplification for 30 s at 94°C, 20 s at 56°C and 30 s at 72°C, and a final elongation step of 72°C for 3 min. PCR products were separated by electrophoresis on a 1% (w/v) agarose gel, and the cDNA fragments of the predicted molecular weight were extracted from the gel using the QIAquick Gel Extraction Kit (Qiagen). Purified amplicons were cloned and sequenced as detailed elsewhere [41].

### Bioinformatic analyses

To ascertain the identity of the cod cDNA sequences obtained, BLASTX searches were performed against the NCBI database (ncbi.nlm.nih.gov). Moreover, *in silico* cloning using the Atlantic cod genome draft (codgenome.no) was performed to obtain longer *mll* and *setd1* sequences for phylogenetic analysis. Putative deduced amino acid sequences were aligned with the corresponding orthologues in various species (Table S1) using MUSCLE (drive5.com). To eliminate gaps and divergent regions, he alignment was trimmed with Gblocks 0.91b (molevol.cmima.csic.es). The resulting multiple sequence alignments were used for bayesian (MrBayes v3.1.2, mrbayes.csit.fsu.edu) and likelihood (PhyML 3.0, www.atgc-montpellier.fr/phyml) phylogenetic analyses. Bayesian phylogenetic trees were obtained using a mixed model of amino acid substitution (1,000,000 generations, sampling every 10^th^ generation and burning the first 10,000 trees) and the likelihood analysis was performed using the LG substitution model with 4 substitution rate categories and an estimated γ shape parameter. Graphical representations of phylogenetic trees were obtained with PhyloWidget (phylowidget.org). Synteny analyses of all *mll* and *setd1* genes were performed on the Genomicus v64.01 genome browser (www.dyogen.ens.fr/genomicus-64.01).

### Semi-quantitative PCR (RT-PCR)

cDNAs were synthesized from total RNA extracted from brain, blood, muscle, gill, head kidney, kidney, heart, liver, spleen, stomach, intestine, immature testes (gonado-somatic index, GSI = 1.9%) and ovaries (GSI = 1.5%) of two-year old Atlantic cod. Semi-quantitative RT-PCR was conducted for each cod *mll* paralogue using the respective qPCR primer sets indicated on Table 1. *Actb* and *eef1a* were used as internal controls. Thermocycling parameters were 94°C for 3 min, followed by 35 cycles of 30 s at 94°C, 30 s at 60°C and 30 s at 72°C, with by a final elongation step of 72°C for 3 min. PCR products were analysed by electrophoresis on a 1% (w/v) agarose gel, visualised and photographed on a Kodak gel documentation system v.4.0.5 (Oslo, Norway).

### Quantitative real-time PCR (qPCR)

Total fast muscle RNA and cDNA was obtained as above from six fish from each of the two different photoperiod groups at the start of the light treatment and 0.5, 1, 7, 30, 60, 120 and 180 days thereafter. Target and reference genes were amplified using the primer sets indicated on Table 1. These primers were designed with the GenScript Real-time PCR software (www.genscript.com) across exon borders determined by Spidey (www.ncbi.nlm.nih.gov/spidey) to avoid amplification of contaminating genomic DNA [42]. Quantification of transcript levels was performed by qPCR using the LightCycler® 480 SYBR Green I Master chemistry (Roche) on a LightCycler® 480 (Roche), as previously described [41]. Fifty-fold diluted muscle cDNA samples were run in duplicate, and minus reverse transcriptase and no template controls were included in the reactions. The PCR reaction was performed at 95°C for 15 min, followed by 45 cycles of 15 s at 94°C, 20 s at 60°C and 20 s at 72°C. Five-point standard curves of a 2-fold dilution series were prepared from pooled RNA in order to calculate amplification efficiencies [42]. Cycle threshold (*C_T_*) values were determined by the LightCycler® 480 software with a fluorescence level arbitrarily set to one. The suitability of β-actin (*actb*), acidic ribosomal protein (*arp*), eukaryotic elongation factor 1α (*eef1a*) and ubiquitin (*ubi*) as reference genes for this experimental setup was investigated [43] and raw target gene data were corrected with geNorm normalisation factors (medgen.ugent.be/∼jvdesomp/genorm/) that corresponded to the geometric average of *arp* and *ubi*, the two most stable genes. Differences in *mll* paralogues, MRFs (*myog* and *myf5*) and *pax7* expression between the two photoperiod groups were examined with the GraphPad Prism software using Mann-Whitney tests, since the data were not normally distributed. Significance levels were set at P<0.05.

## Supporting Information

Figure S1 **Partial synteny map of the genomic region surrounding** ***mll1*** **.** Orthologous genes in *Gadus morhua*, *Oryzias latipes*, *Gasterosteus aculeatus*, *Takifugu rubripes*, *Tetraodon nigroviridis* and *Danio rerio* are colour coded and represented by block arrows that show their orientation in the genome. *Mll1* paralogues are indicated by the arrow.
(TIF)

Figure S2 **Partial synteny map of the genomic region surrounding** ***mll3a*** **.** Orthologous genes in *Gadus morhua*, *Oryzias latipes*, *Gasterosteus aculeatus*, *Takifugu rubripes*, *Tetraodon nigroviridis* and *Danio rerio* are colour coded and represented by block arrows that show their orientation in the genome. *Mll3a* paralogues are indicated by the arrow.
(TIF)

Figure S3 **Partial synteny map of the genomic region surrounding** ***mll3b*** **.** Orthologous genes in *Gadus morhua*, *Oryzias latipes*, *Gasterosteus aculeatus*, *Takifugu rubripes*, *Tetraodon nigroviridis* and *Danio rerio* are colour coded and represented by block arrows that show their orientation in the genome. *Mll3b* paralogues are indicated by the arrow.
(TIF)

Figure S4 **Partial synteny map of the genomic region surrounding** ***mll4a*** **.** Orthologous genes in *Gadus morhua*, *Oryzias latipes*, *Gasterosteus aculeatus*, *Takifugu rubripes*, *Tetraodon nigroviridis* and *Danio rerio* are colour coded and represented by block arrows that show their orientation in the genome. *Mll4a* paralogues are indicated by the arrow.
(TIF)

Figure S5 **Partial synteny map of the genomic region surrounding** ***mll4b*** **.** Orthologous genes in *Gadus morhua*, *Oryzias latipes*, *Gasterosteus aculeatus*, *Takifugu rubripes*, *Tetraodon nigroviridis* and *Danio rerio* are colour coded and represented by block arrows that show their orientation in the genome. *Mll4b* paralogues are indicated by the arrow.
(TIF)

Figure S6 **Partial synteny map of the genomic region surrounding** ***mll5*** **.** Orthologous genes in *Gadus morhua*, *Oryzias latipes*, *Gasterosteus aculeatus*, *Takifugu rubripes*, *Tetraodon nigroviridis* and *Danio rerio* are colour coded and represented by block arrows that show their orientation in the genome. *Mll5* paralogues are indicated by the arrow.
(TIF)

Figure S7 **Partial synteny map of the genomic region surrounding** ***setd1a*** **.** Orthologous genes in *Gadus morhua*, *Oryzias latipes*, *Gasterosteus aculeatus*, *Takifugu rubripes*, *Tetraodon nigroviridis* and *Danio rerio* are colour coded and represented by block arrows that show their orientation in the genome. *Setd1a* paralogues are indicated by the arrow.
(TIF)

Figure S8 **Partial synteny map of the genomic region surrounding** ***setd1ba*** **.** Orthologous genes in *Gadus morhua*, *Oryzias latipes*, *Gasterosteus aculeatus*, *Takifugu rubripes*, *Tetraodon nigroviridis* and *Danio rerio* are colour coded and represented by block arrows that show their orientation in the genome. *Setd1ba* paralogues are indicated by the arrow.
(TIF)

Figure S9 **Partial synteny map of the genomic region surrounding** ***setd1bb*** **.** Orthologous genes in *Gadus morhua*, *Oryzias latipes*, *Gasterosteus aculeatus*, *Takifugu rubripes*, *Tetraodon nigroviridis* and *Danio rerio* are colour coded and represented by block arrows that show their orientation in the genome. *Setd1bb* paralogues are indicated by the arrow.
(TIF)

Table S1 GenBank accession numbers for five *mll* and two *setd1* paralogues and corresponding proteins.
(DOCX)

Table S2 Orthologues of mll and SET domain genes from yeast to human.
(DOCX)
