# Supplementary figures and images for: Photoperiod Influences Growth and mll (Mixed-Lineage Leukaemia) Expression in Atlantic Cod
Source: PLoS One. 2012 May 9;7(5):e36908. doi: 10.1371/journal.pone.0036908 (PMC3348894; doi:10.1371/journal.pone.0036908)

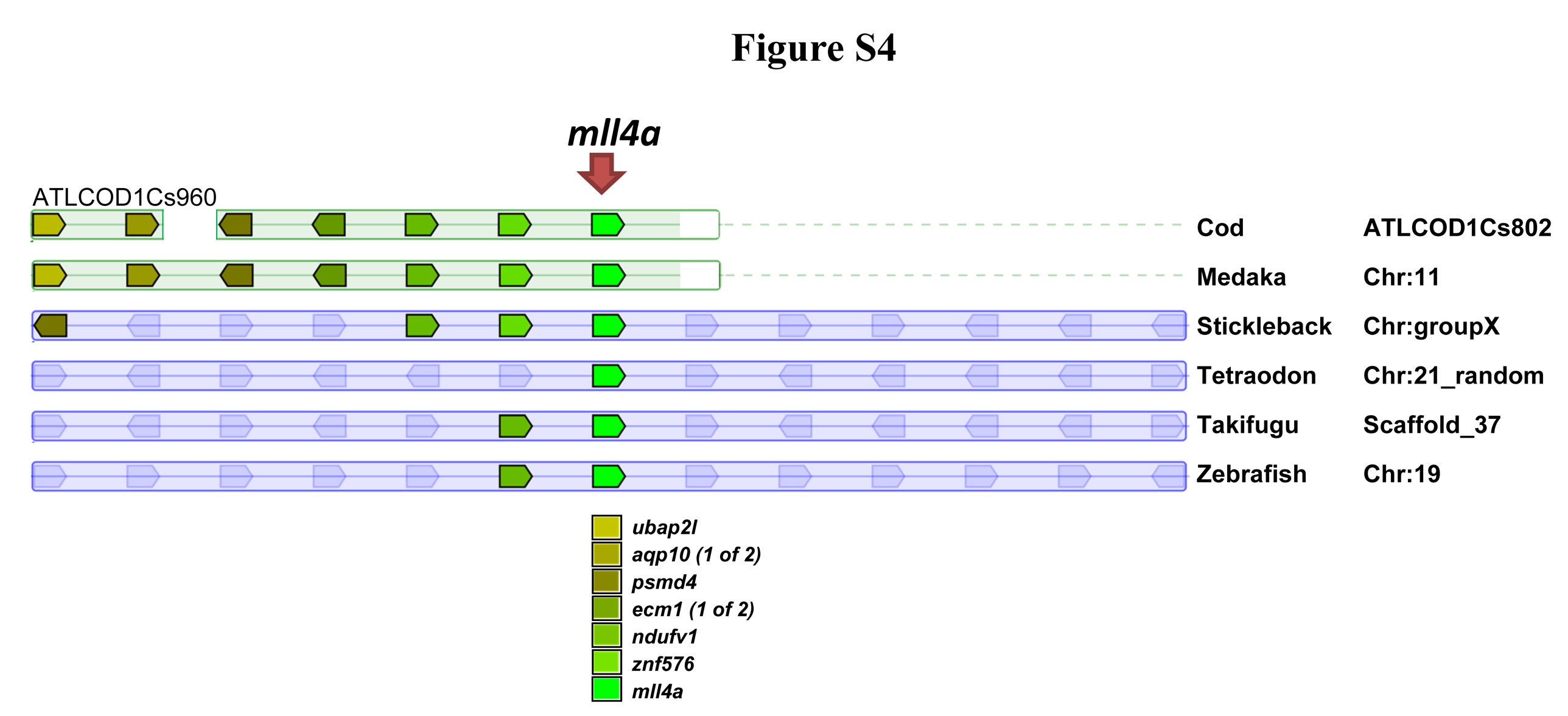

Supplement: Figure S4 — Partial synteny map of the genomic region surrounding mll4a . Orthologous genes in Gadus morhua, Oryzias latipes, Gasterosteus aculeatus, Takifugu rubripes, Tetraodon nigroviridis and Danio rerio are colour coded and represented by block arrows that show their orientation in the genome. Mll4a paralogues are indicated by the arrow. (TIF) [file pone.0036908.s004.tif]

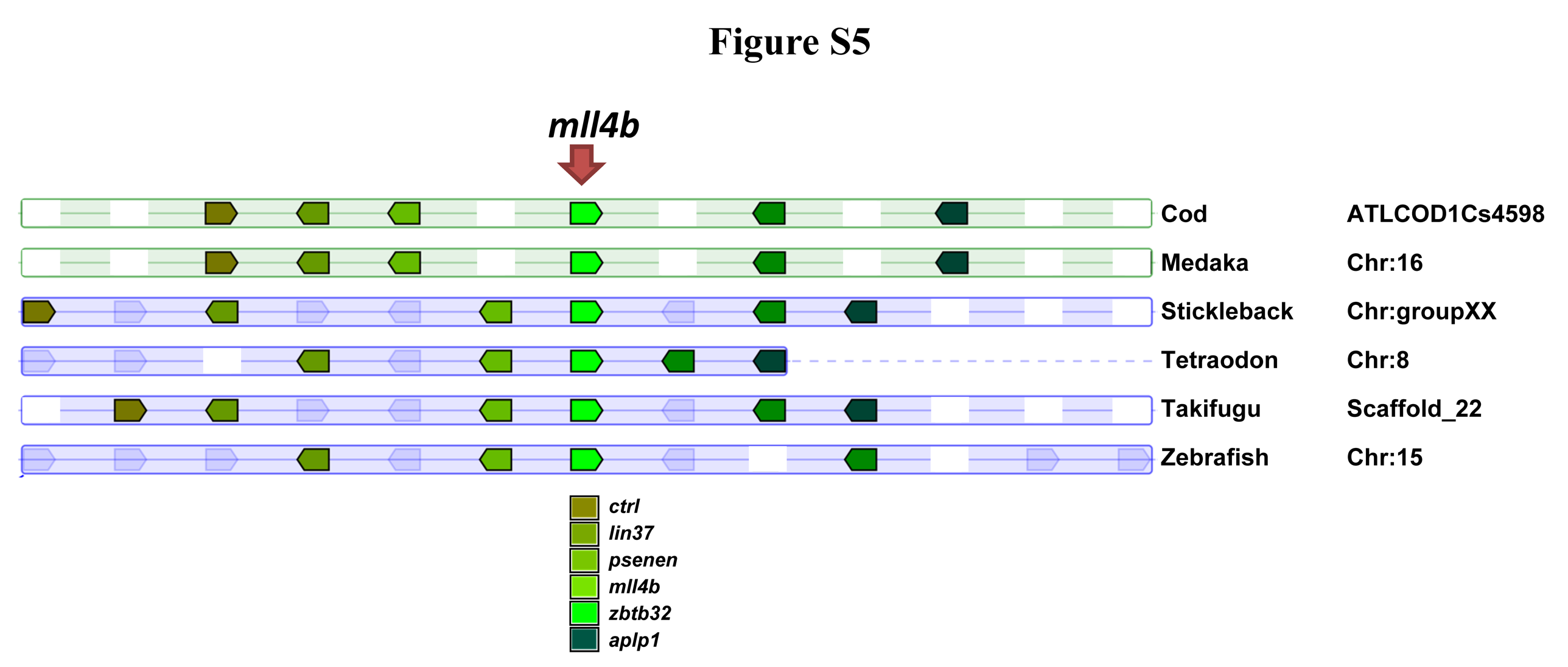

Supplement: Figure S5 — Partial synteny map of the genomic region surrounding mll4b . Orthologous genes in Gadus morhua, Oryzias latipes, Gasterosteus aculeatus, Takifugu rubripes, Tetraodon nigroviridis and Danio rerio are colour coded and represented by block arrows that show their orientation in the genome. Mll4b paralogues are indicated by the arrow. (TIF) [file pone.0036908.s005.tif]
