# Supplementary material for: Photoperiod Influences Growth and mll (Mixed-Lineage Leukaemia) Expression in Atlantic Cod
Source: PLoS One. 2012 May 9;7(5):e36908. doi: 10.1371/journal.pone.0036908 (PMC3348894; doi:10.1371/journal.pone.0036908)
Supplement: Table S1 — GenBank accession numbers for five mll and two setd1 paralogues and corresponding proteins. (DOCX) [file pone.0036908.s010.docx]

Table S1. GenBank accession numbers for five *mll* and two *setd1* paralogues and corresponding proteins.

| **Species** | **Gene** | **mRNA** | **Protein** |
| --- | --- | --- | --- |
| MLL |  |  |  |
| *H.sapiens* | *MLL* | NM_005933.3 | NP_005924.2 |
| *P.troglodytes* | *mll* | XM_508792.2 | XP_508792.2 |
| *C.lupus* | *mll* | XM_536554.2 | XP_536554.2 |
| *B.taurus* | *mll* | XM_585092.3 | XP_585092.3 |
| *M.musculus* | *Mll1* | NM_001081049.1 | NP_001074518.1 |
| *R.norvegicus* | *mll1* | XM_236194.4 | XP_236194.4 |
| *G.gallus* | *mll* | XM_417896.2 | XP_417896.2 |
| *D.rerio* | *mll* | NM_001110279.1 | NP_001103749.1 |
| *G.morhua* | *mll* | GU441836.2 | ADG23060.1 |
| MLL2 |  |  |  |
| *H.sapiens* | *MLL2* | NM_003482.3 | NP_003473.3 |
| *C.lupus* | *mll2* | XM_543684.2 | XP_543684.2 |
| *B.taurus* | *mll2* | XM_583302.4 | XP_583302.4 |
| *M.musculus* | *Mll2* | XM_619357.4 | XP_619357.4 |
| *X.tropicalis* | *mll2* | XM_002935121.1 | XP_002935167.1 |
| *O.niloticus* | *mll2* | XM_003448244.1 | XP_003448292.1 |
| *G.morhua* | *mll2* | GU441837.1 | ADG23061.1 |
| MLL3 |  |  |  |
| *H.sapiens* | *MLL3* | NM_170606.2 | NP_733751.2 |
| *P.troglodytes* | *mll3* | XM_519508.2 | XP_519508.2 |
| *M.musculus* | *Mll3* | NM_001081383.1 | NP_001074852.1 |
| *G.gallus* | *mll3* | XM_418542.2 | XP_418542.2 |
| *O.niloticus* | *mll3* | XM_003437904.1 | XP_003437952.1 |
| *D.rerio* | *mll3a* | XM_001919246.1 | XP_001919281.1 |
| *G.morhua* | *mll3* | GU441838.1 | ADG23062.1 |
| MLL4 |  |  |  |
| *H.sapiens* | *MLL4* | NM_014727.1 | NP_055542.1 |
| *P.troglodytes* | *mll4* | XM_512597.2 | XP_512597.2 |
| *M.musculus* | *Wbp7* | NM_029274.2 | NP_083550.2 |
| *R.norvegicus* | *mll4* | XM_341829.3 | XP_341830.3 |
| *D.rerio* | *mll4a* | XM_684255.5 | XP_689347.3 |
| *D.rerio* | *mll4b* | XM_002664671.1 | XP_002664717.1 |
| *G.morhua* | *mll4* | GU441839.2 | ADG23063.1 |
| MLL5 |  |  |  |
| *H.sapiens* | *MLL5* | NM_182931.2 | NP_891847.1 |
| *C.lupus* | *mll5* | XM_533102.2 | XP_533102.2 |
| *B.taurus* | *mll5* | XM_001789698.1 | XP_001789750.1 |
| *M.musculus* | *Mll5* | NM_026984.1 | NP_081260.1 |
| *R.norvegicus* | *mll5* | XM_231287.4 | XP_231287.4 |
| *D.rerio* | *mll5* | XM_687873.2 | XP_692965.2 |
| *G.morhua* | *mll5* | GU441840.1 | ADG23064.1 |
| SETD1A |  |  |  |
| *H.sapiens* | *SETD1A* | NM_014712.1 | NP_055527.1 |
| *P.troglodytes* | *setd1a* | XM_523492.2 | XP_523492.2 |
| *B.taurus* | *setd1a* | XM_001251264.1 | XP_001251265.1 |
| *M.musculus* | *Setd1a* | NM_178029.3 | NP_821172.2 |
| *D.rerio* | *setd1a* | XM_001920817.1 | XP_001920852.1 |
| *C.elegans* | *set-2* | NM_065639.3 | NP_498040.1 |
| *G.morhua* | *setd1a* | GU441841.1 | ADG23065.1 |
| SETD1B |  |  |  |
| *H.sapiens* | *SETD1B* | NM_015048.1 | NP_055863.1 |
| *C.lupus* | *setd1b* | XM_543382.2 | XP_543382.2 |
| *B.taurus* | *setd1b* | XM_591902.4 | XP_591902.4 |
| *M.musculus* | *Setd1b* | NM_001040398.1 | NP_001035488.1 |
| *G.gallus* | *setd1b* | NM_001030661.1 | NP_001025832.1 |
| *D.rerio* | *setd1ba* | NM_001045134.2 | NP_001038599.2 |
| *D.rerio* | *setd1bb* | XM_694848.5 | XP_699940.5 |
| *G.morhua* | *setd1b* | HQ315825.1 | ADO84691.1 |
