# Supplementary material for: Photoperiod Influences Growth and mll (Mixed-Lineage Leukaemia) Expression in Atlantic Cod
Source: PLoS One. 2012 May 9;7(5):e36908. doi: 10.1371/journal.pone.0036908 (PMC3348894; doi:10.1371/journal.pone.0036908)
Supplement: Table S2 — Orthologues of mll and SET domain genes from yeast to human. (DOCX) [file pone.0036908.s011.docx]

Table S2. Orthologues of *mll* and SET domain genes from yeast to human (Modified from Sun, 2008). Isoforms in fish species were determined based on the combined information in the Genbank database and syntheny analysis with *Genomicus*.

| ***S. cerevisiae*** | ***C. elegans*** | ***D. melanogaster*** | ***D. rerio*** | ***O. latipes*** | ***G. aculeatus*** | ***T. rubripes*** | ***T. nigroviridis*** | ***X. laevis*** | ***G. gallus*** | ***M. musculus*** | ***H. sapience*** | **HMT activity** |
| --- | --- | --- | --- | --- | --- | --- | --- | --- | --- | --- | --- | --- |
|  | ***tag-350*** | ***trr*** | ***mll2*** | ***mll2*** | ***mll2*** | ***mll2*** | ***mll2*** | ***mll2*** | ***mll2*** | ***Mll2*** | ***MLL2*** | **H3K4** |
|  |  |  | ***mll3a*** | ***mll3a*** | ***mll3a*** | ***mll3a*** | ***mll3a*** | ***mll3*** | ***mll3*** | ***Mll3*** | ***MLL3*** |  |
|  |  |  | ***mll3b*** | ***mll3b*** | ***mll3b*** | ***mll3b*** | ***mll3b*** |  |  |  |  |  |
|  |  | ***trx*** | ***mll4a*** | ***mll4a*** | ***mll4a*** | ***mll4a*** | ***mll4a*** | ***mll4*** | **no data** | ***Mll4*** | ***MLL4*** |  |
|  |  |  | ***mll4b*** | ***mll4b*** | ***mll4b*** | ***mll4b*** | ***mll4b*** |  |  |  |  |  |
|  |  |  | ***mll*** | ***mll*** | ***mll*** | ***mll*** | ***mll1a*** | ***mll*** | ***mll*** | ***Mll*** | ***MLL*** | **H3K4** |
|  |  |  |  |  |  |  | ***mll1b*** |  |  |  |  |  |
|  | ***set-24*** | ***GC9007*** | ***mll5*** | ***mll5*** | ***mll5*** | ***mll5*** | ***mll5*** | ***mll5*** | ***mll5*** | ***Mll5*** | ***MLL5*** |  |
|  | ***set-9*** |  |  |  |  |  |  |  |  |  |  |  |
|  | ***set-26*** |  |  |  |  |  |  |  |  |  |  |  |
| ***SET1*** | ***set-2*** | ***CG40351*** | ***setd1a*** | ***setd1a*** | ***setd1a*** | ***setd1a*** | ***setd1a*** | ***setd1a*** | **no data** | ***Setd1a*** | ***SETD1A*** | **H3K4** |
|  |  |  | ***setd1ba*** | ***setd1ba*** | ***setd1ba*** | ***setd1ba*** | ***setd1ba*** | ***setd1b*** | ***setd1b*** | ***Setd1b*** | ***SETD1B*** |  |
|  |  |  | ***setd1bb*** | ***setd1bb*** | ***setd1bb*** | ***setd1bb*** | ***setd1bb*** |  |  |  |  |  |
